# Capsaicin consumption reduces brain amyloid-beta generation and attenuates Alzheimer’s disease-type pathology and cognitive deficits in APP/PS1 mice

**DOI:** 10.1038/s41398-020-00918-y

**Published:** 2020-07-13

**Authors:** Jun Wang, Bin-Lu Sun, Yang Xiang, Ding-Yuan Tian, Chi Zhu, Wei-Wei Li, Yu-Hui Liu, Xian-Le Bu, Lin-Lin Shen, Wang-Sheng Jin, Zhen Wang, Gui-Hua Zeng, Wei Xu, Li-Yong Chen, Xiao-Wei Chen, Zhian Hu, Zhi-Ming Zhu, Weihong Song, Hua-Dong Zhou, Jin-Tai Yu, Yan-Jiang Wang

**Affiliations:** 1 Department of Neurology and Center for Clinical Neuroscience, Daping Hospital, Third Military Medical University, 400042 Chongqing, China; 2 Department of Neurology, Sichuan Academy of Medical Sciences & Sichuan Provincial People’s Hospital, 610072 Chengdu, China; 3 Shigatse Branch, Xinqiao Hospital, Third Military Medical University, 857000 Shigatse, China; 4 Department of anaesthesiology, Daping Hospital, Third Military Medical University, 400042 Chongqing, China; 5 Department of Neurology, Qingdao Municipal Hospital, Qingdao University, 266071 Qingdao, China; 6 Brain Research Center, Third Military Medical University, 400038 Chongqing, China; 7 Department of Physiology and Institute of Brain and Intelligence, Third Military Medical University, 400038 Chongqing, China; 8 Center for Hypertension and Metabolic Diseases, Department of Hypertension and Endocrinology, Daping Hospital, Third Military Medical University, 400042 Chongqing, China; 9 Townsend Family Laboratories, Department of Psychiatry, Brain Research Center, The University of British Columbia, Vancouver, BC V6T 1Z3 Canada; 10 Department of Neurology, Huashan Hospital, Fudan University, 200040 Shanghai, China; 11 Chongqing Key Laboratory of Ageing and Brain Diseases, Daping Hospital, Third Military Medical University, 400042 Chongqing, China; 12 Center for Excellence in Brain Science and Intelligence Technology, Chinese Academy of Sciences, 200031 Shanghai, China

**Keywords:** Neuroscience, Diseases

## Abstract

Alzheimer’s disease (AD) is the most common cause of age-related dementia and is currently incurable. The failures of current clinical trials and the establishment of modifiable risk factors have shifted the AD intervention from treatment to prevention in the at-risk population. Previous studies suggest that there is a geographic overlap between AD incidence and spicy food consumption. We previously reported that capsaicin-rich diet consumption was associated with better cognition and lower serum Amyloid-beta (Aβ) levels in people aged 40 years and over. In the present study, we found that intake of capsaicin, the pungent ingredient in chili peppers, reduced brain Aβ burden and rescued cognitive decline in APP/PS1 mice. Our in vivo and in vitro studies revealed that capsaicin shifted Amyloid precursor protein (APP) processing towards α-cleavage and precluded Aβ generation by promoting the maturation of a disintegrin and metalloproteinase 10 (ADAM10). We also found that capsaicin alleviated other AD-type pathologies, such as tau hyperphosphorylation, neuroinflammation and neurodegeneration. The present study suggests that capsaicin is a potential therapeutic candidate for AD and warrants clinical trials on chili peppers or capsaicin as dietary supplementation for the prevention and treatment of AD.

## Introduction

Alzheimer’s disease (AD), the most common cause of age-related dementia, causes heavy social and economic burdens. There is no effective therapy to cure AD or even to halt the course of the disease. With the failure of a series of clinical trials, the strategy of AD treatment has recently shifted to disease prevention, with clinical testing carried out in at-risk populations^1–5^. In fact, our analysis suggests that one-third of AD cases worldwide are attributable to several common modifiable risk factors^6–8^, most of which can be controlled by healthy dietary and lifestyle, making dietary and lifestyle intervention research hotspots^9,10^.

Chili pepper is a basic element of culinary culture consumed worldwide as vegetable and spice. Capsaicin is the major component in chili pepper, accounting for the spicy/pungent flavour. Previous studies suggest that there is an interesting geographic overlap between AD incidence and spicy food consumption in China. The incidence of AD in west China (3.99/1000 person-years) is lower than that in the east (5.58/1000 person-years)^11^, and in the west, the proportion of dishes with chili is higher and the pungency degree is greater than in the east^12,13^. Previously, we reported that capsaicin-rich diet consumption was associated with better cognition and lower serum amyloid-β (Aβ) levels in people aged 40 years and over^14^. These findings imply that capsaicin may be protective against AD.

In the present study, we aimed to further investigate whether capsaicin plays protective roles in AD pathogenesis. We investigated the effects of capsaicin on brain Aβ burden and cognition in APPswe/PS1dE9 (APP/PS1) mice, and explored the underlying mechanisms in human neuroblastoma SH-SY5Y-APP695 cells and APP/PS1 mice. Additionally, we observed the effects of capsaicin on other AD-type pathologies, including tau hyperphosphorylation, neuroinflammation, and neurodegeneration in APP/PS1 mice.

## Methods

### Cell culture and treatment

Human neuroblastoma SH-SY5Y-APP695 cells were stably transfected with the human wild-type APP695^15^. The cells were cultured in Dulbecco’s modified Eagle’s medium (DMEM) containing 4 mM l-glutamine and 4500 mg/L glucose (HyClone, USA) at 37°C and 5% CO_2_. The medium was supplemented with 10% foetal bovine serum (FBS, HyClone, USA). Zeocin^TM^ selection antibiotic (Invitrogen, USA) were added (100 μg/ml) to avoid bacterial contamination and mycoplasma contamination tests were conducted before analysis. To observe the dose-effect relationship between capsaicin and Aβ generation, the cells were incubated without (control) or with various concentrations of capsaicin (0.1, 1, 5, 10, 50 μM) (Sigma-Aldrich, USA) for 24 h. The culture medium did not contain FBS when the cells were treated with capsaicin. The cell culture medium of each group was subjected to ELISA for Aβ_40_ and Aβ_42_ concentrations, while the cell lysates were subjected to Western blot for APP metabolites and ELISA for Aβ_40_ and Aβ_42_ concentrations in triplicate.

### MTT assay

To evaluate the toxicity of capsaicin, the cells were seeded in 96-well plates (5 × 10^4^ cells/ml) and incubated without (control) or with various concentrations of capsaicin (5, 10, 25, 50, 75, 100, 125, 150 μM) for 24 h. The cell culture medium was changed immediately before the MTT [3-(4,5-dimethylthiazol-2-yl)-2,5-diphenyltetrazolium bromide] assay to avoid an interaction between MTT and capsaicin. Then 10 μl MTT (5 mg/ml, Sigma-Aldrich, USA) solution was added for 4 h. The supernatant was then removed, and 100 μl DMSO was added to dissolve the dye crystals. Absorbance at 490 nm was read. Capsaicin impaired cell viability at concentrations >75 μM. At the concentration of 75 μM, cell viability presented impaired tendency **(**Supplemental Fig. 1).

### Animals and treatment

All mouse husbandry procedures and operations in the present study were approved by the Third Military Medical University Animal Welfare Committee. The APP/PS1 transgenic (Tg) mice on C57BL/6 background and C57BL/6 wild-type (Wt) mice were obtained from Jackson Laboratory and bred in the animal facility of Daping Hospital. The mice were housed under a 12 h light/dark cycle with free access to food and water. Tg mice were given the normal standard chow (Tg control groups) or normal chow plus with 0.01% capsaicin^16,17^ (Tg cap group) at randomization from 3 months old to 9 months old (*n* = 8 per group, half female and half male). Based on the amount of consumed food per day and the weight of mice, we approximately estimated that the daily intake of capsaicin was about 30 mg/kg. Age- and sex-matched WT mice were used as baseline controls.

### Behavioural tests

A battery of behavioural tests comprising the Morris water maze (MWM), Y-maze and open-field tests were conducted to assess behavioural performance of participant mice as previously described^18,19^. In brief, the MWM test was conducted in a circular pool with 120 cm in diameter, which was filled with opaque water stained with milk and surrounded by a set of spatial cues. The tank was imaginarily divided into four quadrants. A platform with 9 cm diameter was submerged 1 cm under water surface in a quadrant. The MWM test consisted of three platform trials per day for 5 consecutive days, followed by a probe trial. In the platform trial, the mouse navigated in the pool to locate the platform and was then able to escape. If the mouse failed to locate the platform within 60 s, it was directed to the platform. The mouse was allowed to remain on the platform for 60 s once it escaped onto the platform. The distance of the path taken and the escape latency were measured to test spatial learning ability. In the probe trial, the platform was withdrawn. The mouse navigated in the pool freely for 1 min. The time spent in each quadrant and the number of annulus crossings were recorded to assess memory consolidation. The Y-maze tests were comprised of the spontaneous alternation test and novel arm exploration test. The Y-maze apparatus consists of three enclosed yellow Plexiglas arms (31 cm long, 8 cm wide and 31 cm high) at 120° angles to each other, radiating out from the central point. In the spontaneous alternation test, the mouse was placed at the end of one arm facing the wall and allowed to walk freely in the maze for a 5 min session. Alternation, aiming to test spatial working memory, was defined as consecutive entries into the three arms on overlapping triplet sets without repetition. In the novel arm exploration test, the mouse was placed at the end of home arm and allowed to explore the maze for 5 min with one arm (novel arm) closed in the training trial. The mouse was returned to their home cage until the retrieval trial, during which the mouse was allowed to explore all three arms freely within the maze for 5 min. The percentage of novel arm entry and time spent in the novel arm were analyzed to measure spatial recognition memory. In the open-field test, the mouse was placed in the centre of the open-field apparatus (50 cm length, 50 cm wide and 45 cm high), and allowed to explore freely for 5 min. The total travelling distance was recorded to measure the spontaneous locomotor activity, whereas the ratio of time in the central zone to the peripheral zone and the numbers of rearings, groomings, defecations, and urinations were recorded to determine anxiety-like behaviour. All performances were video-recorded and analyzed with image-analyzing software (ANY-maze, Stoelting, Wood Dale, IL, USA).

### Brain sampling

The mice were sacrificed at 9 months old. There was no significant difference in body weight of mice among each group. Blood was drawn from the eyes, followed by intracardial perfusion with 100 ml of 0.1% NaNO_2_ in normal saline under anaesthesia. Brains were sampled as described previously^20^. Briefly, the left hemisphere was fixed with 4% paraformaldehyde, and coronal sections were cut at 35-μm thickness for histological analysis, while the right hemisphere was snap-frozen and ground into powder in liquid nitrogen, divided into three vials, weighed, and stored at −80 °C for biochemical analysis. Brain sampling and histological/biochemical analysis were conducted by different investigators for blinding.

### Histology and quantification

#### Aβ plaques and cerebral amyloid angiopathy (CAA)

For Aβ plaques in parenchyma, brain tissue sections were stained with Congo red for compact Aβ plaques or with 6E10 antibody using a free-floating immunohistochemistry (IHC) method for total Aβ plaques containing compact and diffuse plaques^20^. The area fraction and plaque number of Congo red- or 6E10-positive staining in neocortex and hippocampus were quantified with ImageJ software. CAA was visualized with Congo red staining and manually selected from two slices of the hippocampus^21^, CAA number per slice were quantified.

In histology and quantification, a series of five equally spaced tissue sections (~1.3 mm apart) spanning the entire brain of each mouse were used for immunohistochemistry staining (Supplemental Fig. 2**)**. All the sections were stained and photos were taken under the same conditions at the same time. Images were analyzed with ImageJ software under the same conditions in a blinded manner to the group information of the sections.

#### Tau pathology, neuroinflammation, and neurodegeneration

Immunohistochemistry was used to detect phosphorylated tau with anti-pS396-tau antibody (Signalway, USA), microgliosis with anti-CD68 antibody (Abcam, UK), and astrocytosis with anti-glial fibrillary acidic protein (GFAP) antibody (Abcam, UK). Apoptosis of neurons was measured by double-immunofluorescence staining for NeuN (Abcam, UK) and caspase-3 (Millipore, USA). Neuronal loss and neurite degeneration were detected by double-immunofluorescence staining for NeuN and microtubule-associated protein (MAP)-2 (Millipore, USA). The fraction of positive staining as a proportion of total area and integrated fluorescence intensity were quantified with ImageJ software.

### ELISA

One vial of brain powder was used for sequential protein extraction in Tris buffer solution (TBS), 2% sodium dodecyl sulfonate (SDS) and 70% formic acid (FA). The levels of human Aβ42 and Aβ40 in the TBS, SDS, and FA were measured using ELISA kits (Invitrogen, USA) according to the manufacturer’s instructions. Inflammatory factors, including mouse tumour necrosis factor-alpha (TNF-α), interferon-γ (IFN-γ), interleukin-1β (IL-1β) and IL-6, in brain homogenates were measured using corresponding ELISA kits (R&D Systems, USA).

### RNA extraction and quantitative real-time PCR

The total RNA was extracted from brain powder using TRIzol reagent (Life Technologies, USA) according to the manufacturer’s instructions. For each RNA sample, an equal amount of total RNA (1 μg) was reverse-transcribed into cDNA using iScript^TM^ cDNA synthesis kit (BIO-RAD, USA). qRT-PCR was performed on a CFX96^TM^ Real-Time System (BIO-RAD, USA) with cDNA (equivalent to 50 ng RNA per 20 μl PCR assay).

### Western blot

For Western blot analysis, one vial of brain powder was suspended in RIPA buffer, and proteins were extracted. Identical amounts of RIPA-extracted protein were loaded and separated by 4-20% PAGE Gels (KeyGEN BioTECH, China) and transferred to nitrocellulose membranes. The blots were probed with the following primary antibodies: anti-APP C-terminal antibody (Millipore, USA) to detect C-terminal fragment (CTF)-α and CTF-β, 6E10 (BioLegend, USA) to detect Aβ, full-length APP (APPfl), and secreted APP (sAPP)-α (sAPPα); anti-a disintegrin and metalloproteinase 10 (ADAM10) antibody (Abcam, UK); anti-β-secretase 1 (BACE-1) antibody (Abcam, UK); anti-Presenilin-1 (PS-1) antibody (Abcam, UK); anti-transient receptor potential vanilloid 1 (TRPV1) antibody (Millipore, USA); anti-peroxisome proliferator-activated receptor α (PPARα) antibody (Abcam, UK); anti-insulin-degrading enzyme (IDE) antibody (Millipore, USA); anti-neprilysin (NEP) antibody (Millipore, USA); anti-receptor for advanced glycation end products (RAGE) antibody (Millipore, USA); anti-lipoprotein receptor-related protein 1 (LRP-1) antibody (Abcam, UK); anti-pS199-tau, anti-pS396-tau, anti-pT231-tau, Tau5 antibodies (Signalway, USA); anti-Synapsin-1 (SYN1) antibody (Millipore, USA); anti-postsynaptic density protein 95 (PSD95) antibody (Millipore, USA); anti-synaptosomal associated protein 25 (SNAP25) antibody (Millipore, USA); anti-vesicle-associated membrane protein 1 (VAMP1) antibody (Abcam, UK); anti-β-actin antibody (Sigma, USA). The membranes were incubated with IRDye 800 CW secondary antibodies (Li-COR, USA) and scanned using the Odyssey fluorescent scanner. The band density was normalized to β-actin for analysis.

### Statistics

The results are presented as the mean ± SEM unless otherwise stated. Statistical comparisons between two groups were made using Student’s *t* test or the Mann–Whitney *U* test, as applicable. One-way ANOVA and Tukey’s test were used to compare three groups, and two-way ANOVA was used to compare two groups at multiple timepoints. *P* values less than 0.05 (two-sided) were considered significant. All analyses were performed with GraphPad Prism software, version 7.0, or SPSS software, version 20.0.

## Results

### Dietary capsaicin rescues cognition impairment in APP/PS1 mice

**Fig. 1:**
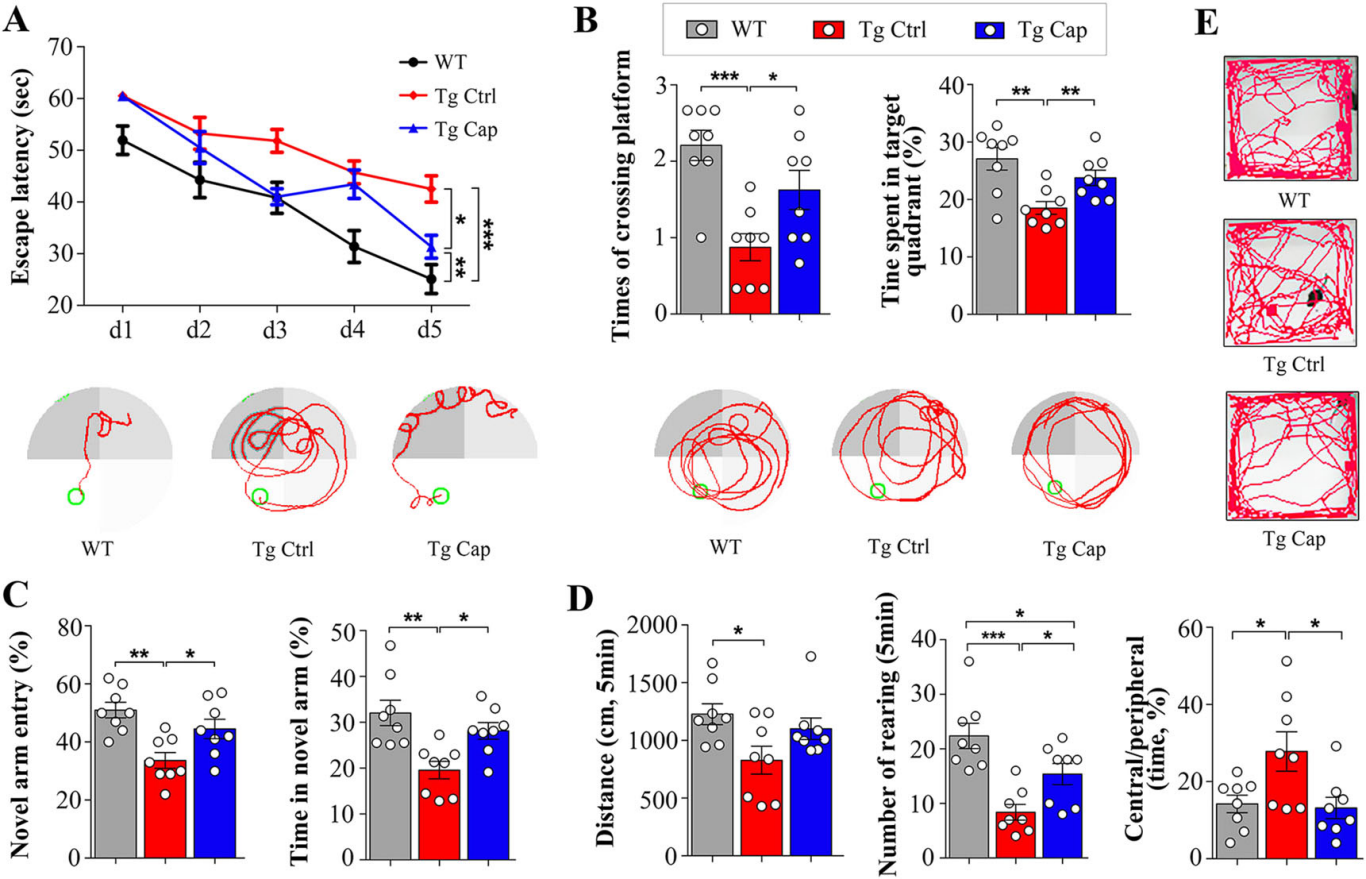
Capsaicin improves behavioural performances of APP/PS1 mice. **a** Escape latency during platform trials in Morris water maze (two-way ANOVA) and representative tracing graphs in platform trials in Morris water maze. **b** Times of crossing platform and time spent in target quadrant in probe test in Morris water maze (one-way ANOVA followed by Turkey’s test), and representative tracing graphs in probe test in Morris water maze. **c** Percentage of novel arm entries and time spent in novel arm in Y-maze test. **d** Distance travelled, number of rearings and the ratio of time spent in central and peripheral areas in open-field test (one-way ANOVA followed by Turkey’s test). **e** Representative tracing graphs in open-field test. *N* = 8 per group. Values are presented as the mean ± SEM. *p < 0.05, **p < 0.01, ***p < 0.001, two-sided. Tg APP/PS1 transgenic mice, WT wild-type mice, Cap Capsaicin, Ctrl Control.

To investigate the effects of dietary capsaicin on cognition impairment in AD, we conducted capsaicin prevention experiments in APP/PS1 mice. The mice were feed with 0.01% capsaicin-rich diet or standard diet from 3 months of age when Aβ pathologies are not formed, and were subjected to analysis at 9 months old, when extensive Aβ pathologies and obvious cognitive impairment occur. APP/PS1 control mice displayed an impaired learning ability and spatial reference memory compared with Wt mice, while the mice received capsaicin diet presented improved spatial learning ability, as reflected by a significant reduction in the escape latency, and better memory consolidation, reflected by a higher number of platform area crossings and more time spent in the target quadrant compared with APP/PS1 controls in the MWM test (Fig. 1a, b). There was no difference in swimming speed during platform trials or path length to the platform during probe trials among each groups (Supplemental Fig. 3a, b). APP/PS1 mice received the capsaicin diet also showed more entries into and more time spent in the novel arm in the Y-maze test, a reflection of better spatial recognition memory (Fig. 1c). However, the mice did not display different performance in the spontaneous exploration test of Y-maze (Supplemental Fig. 3c, d). In open-field tests, the capsaicin-treated mice showed a longer travelling distance, a higher number of rearing and a reduced ratio of time spent in the central zone to the peripheral zone, suggesting an enhanced locomotor activity and anxiety-like behaviour (Fig. 1d, e). The above findings indicate that dietary capsaicin can protect against cognitive decline in APP/PS1 mice.

### Dietary capsaicin attenuates Aβ pathologies in APP/PS1 mice

**Fig. 2:**
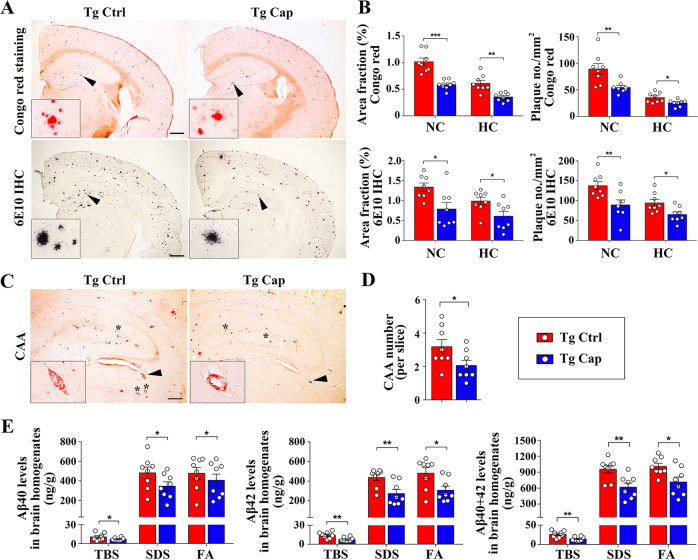
Capsaicin ameliorates amyloid deposition in APP/PS1 mice. **a** Representative images of Congo red staining and 6E10 immunohistochemical (IHC) staining in Tg Ctrl and Tg Cap groups. *Insets* show the representative plaque at higher magnification. *Scale bar*, 500 μm. **b** Comparison of 6E10- or Congo red-positive plaques in the neocortex (NC) and hippocampus (HC). **c** Representative image of cerebral amyloid angiopathy (CAA) visualized using Congo red staining. *Insets* show the representative morphology of CAAs at higher magnification. Other CAAs are marked with a black star. *Scale bar*, 200 μm. **d** Comparison of CAA numbers between Tg Ctrl and Tg Cap mice. **e** Comparison of Aβ_40_, Aβ_42_ and total Aβ levels measured with ELISA in TBS, SDS and formic acid (FA) fractions of brain homogenates. Aβ levels are normalized to the weight of brain tissue. *N* = 8 per group, Student’s *t*-test, two-sided. *p < 0.05, **p < 0.01, ***p < 0.001. Bars express mean ± SEM. Tg APP/PS1 transgenic mice, Cap Capsaicin, Ctrl Control.

We investigated whether capsaicin treatment before Aβ plaque formation would inhibit Aβ deposition in the brain of APP/PS1 mice. We performed Aβ IHC staining (6E10) for total Aβ plaques and Congo red staining for compact Aβ plaques. Compared with APP/PS1 controls, the mice treated with capsaicin displayed significant reductions in area fraction and plaque density of both total and compact plaques in neocortex and hippocampus (Fig. 2a, b). In addition, Congo red staining showed that Aβ deposition in vessel walls, which reflects the formation of CAA, decreased in mice treated with capsaicin (Fig. 2c, d). Consistent with these data, ELISA assays showed significantly lower levels of Aβ_42_, Aβ_40_ and total Aβ in the TBS (extracellular soluble Aβ), SDS (intracellular soluble Aβ) and formic acid (insoluble Aβ) fractions of brain homogenate extracts in mice treated with capsaicin relative to controls (Fig. 2e). Based on the ELISA results, we calculated that dietary capsaicin reduced total Aβ burden by 32.3% in the brain of APP/PS1 mice. Taken together, our findings indicate that dietary capsaicin has preventive potential for AD.

### Capsaicin inhibits Aβ generation via promoting non-amyloidogenic processing of APP

**Fig. 3:**
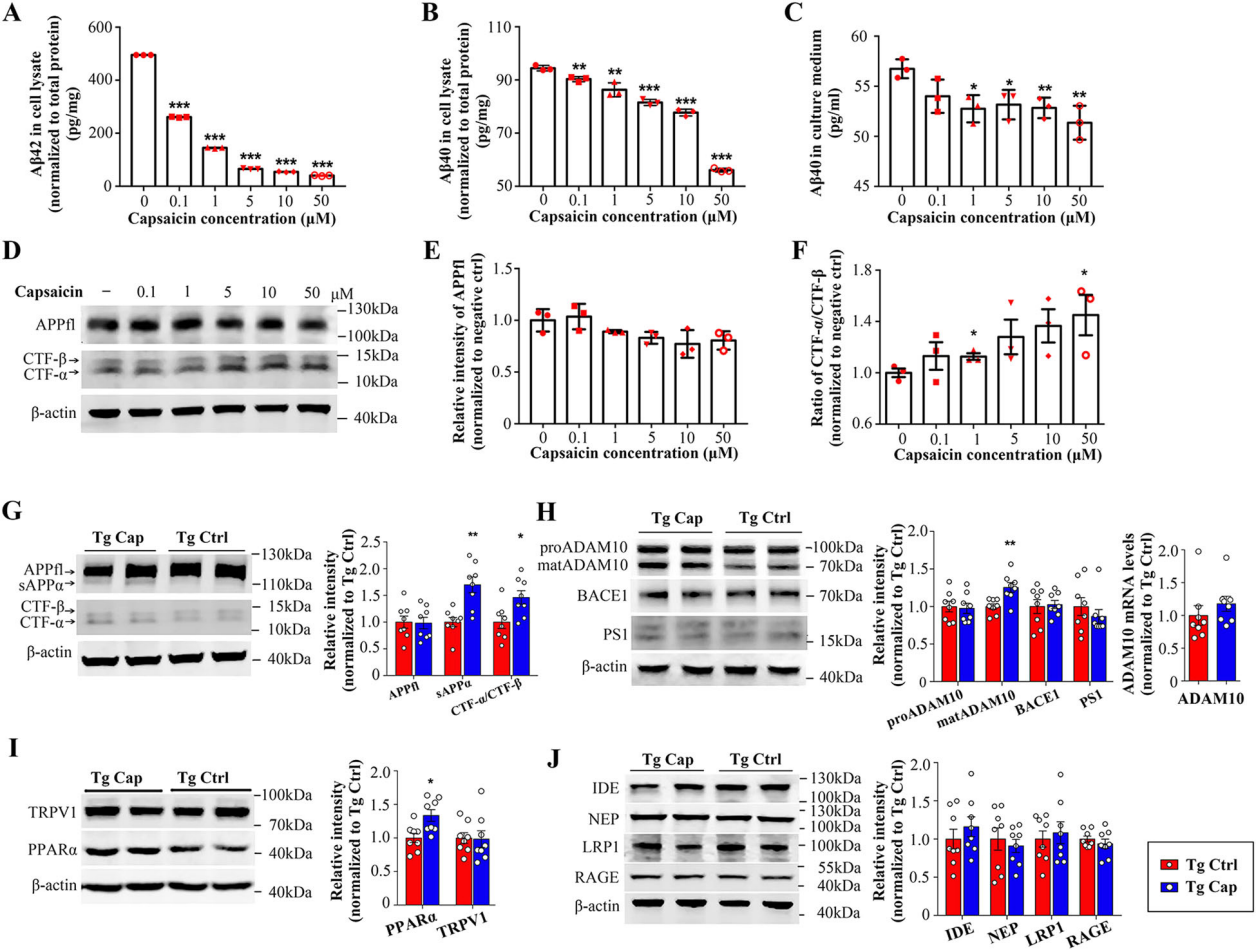
Effects of capsaicin on Aβ metabolism. **a**–**c** Aβ_42_ and Aβ_40_ levels in cell lysates and Aβ_40_ levels in culture medium measured with ELISA in SH-SY5Y-APP695 cells treated with or without various concentrations of capsaicin for 24 h. (*N* = 3, mean ± SEM; Student’s *t* test, two-sided. **p < 0.01, ***p < 0.001). **d** Western blots for full-length APP (APPfl), CTF-α and CTF-β in SH-SY5Y-APP695 cells treated with or without various concentrations of capsaicin for 24 h. **e**, **f** Quantitative analysis of APPfl and the ratio of CTF-α/CTF-β in SH-SY5Y-APP695 cell lysates measured by Western blot (*N* = 3, mean ± SEM; Student’s *t* test, two-sided. *p < 0.05). **g** Western blots and quantitative analysis for APP and APP metabolites in brain homogenates of Tg Ctrl and Tg Cap mice (*N* = 8 per group, mean ± SEM; Student’s *t* test, two-sided. *p < 0.05, **p < 0.01). **h** Western blots and quantitative analysis of APP cleavage enzymes and ADAM10 mRNA levels quantified by qRT-PCR in brain homogenates (*N* = 8 per group, mean ± SEM; Student’s *t* test, two-sided. **p < 0.01). **i** Western blots and quantitative analysis of PPARα and TRPV1 in brain homogenates of Tg Ctrl and Tg Cap mice (*N* = 8 per group, mean ± SEM; Student’s *t* test, two-sided. *p < 0.05). **j** Western blots and quantitative analysis of Aβ-degrading and Aβ-transporting receptors in the blood-brain barrier in brain homogenates (*N* = 8 per group, mean ± SEM; Student’s *t* test, two-sided.). Tg APP/PS1 transgenic mice, Cap Capsaicin, Ctrl Control.

To explore the mechanism underlying Aβ reduction by capsaicin, SH-SY5Y-APP695 cells, which are human neuroblastoma cells overexpressing human wild-type APP695, were treated with or without various concentrations of capsaicin. Aβ is derived from sequential cleavage of APP by β-secretase and γ-secretase within neurons, and then secreted to extracellular space^22^. First, we measured Aβ_40_ and Aβ_42_ levels in cell lysates and culture medium. The results showed that capsaicin treatment decreased levels of both Aβ_40_ and Aβ_42_ in SH-SY5Y-APP695 cell lysates in a dose-dependent manner (Fig. 3a, b). Aβ_40_ levels in culture medium were also dose-dependently reduced by capsaicin **(**Fig. 3c**)**, whereas Aβ_42_ in culture medium was undetectable due to the low concentration (data not shown). Next, we examined the levels of full-length APP and products of APP processing in cell lysates. We found that capsaicin did not affect the expression of full-length APP, but dose dependently increased the ratio of CTF-α/CTF-β (Fig. 3d–f). Our in vitro results suggest that capsaicin inhibits Aβ generation via shifting APP processing towards α-cleavage.

Next, to verify the effects of capsaicin on APP processing, we measured APP and its processing products in brain homogenates of APP/PS1 mice. Consistent with in vitro results, capsaicin did not affect the expression of full-length APP (Fig. 3g). We found that sAPPα and the ratio of CTF-α/CTF-β significantly increased in the brain of mice treated with capsaicin compared with controls (Fig. 3g). Our in vivo results support that capsaicin inhibits Aβ production via promoting non-amyloidogenic processing of APP.

To further verify above findings, we measured levels of secretases responsible for APP processing. ADAM10 is the major α-secretase that catalyses α-cleavage and promotes non-amyloidogenic processing of APP. ADAM10 is first generated as an inactive proenzyme (proADAM10) and matures into an active protease after the removal of its prodomain^23,24^. We found that capsaicin treatment significantly increased brain levels of matADAM10 relative to APP/PS1 controls but did not affect proADAM10 protein or mRNA expression (Fig. 3h), suggesting that capsaicin increases the level of ADAM10 by promoting its maturation or activation. However, no significant differences were observed in BACE1 or PS1 responsible for amyloidogenic processing of APP (Fig. 3h).

It has been reported that activation of PPARα, a transcription factor regulating genes involved in fatty acid metabolism, could stimulate ADAM10-mediated proteolysis of APP^25^. We measured PPARα levels in the brain of APP/PS1 mice and found that PPARα levels increased in the brain of mice treated with capsaicin compared with controls (Fig. 3i), implying that capsaicin may activate ADAM10-mediated APP processing via upregulating PPARα, and thus precluding Aβ generation. We also detected the levels of TRPV1, which is known as capsaicin receptor. Unfortunately, we did not observe significant differences in TRPV1 expression between two groups (Fig. 3i).

Additionally, we tested Aβ clearance-related molecules, including the Aβ-degrading enzymes IDE and NEP, and the Aβ transporters LRP-1 and RAGE, across the blood–brain barrier (BBB). None of these proteins showed significant differences between the two groups (Fig. 3j). Our findings indicate that capsaicin shifts APP processing towards α-cleavage and precludes Aβ generation by promoting the maturation of ADAM10.

### Dietary capsaicin attenuates other AD-type pathologies in APP/PS1 mice

**Fig. 4:**
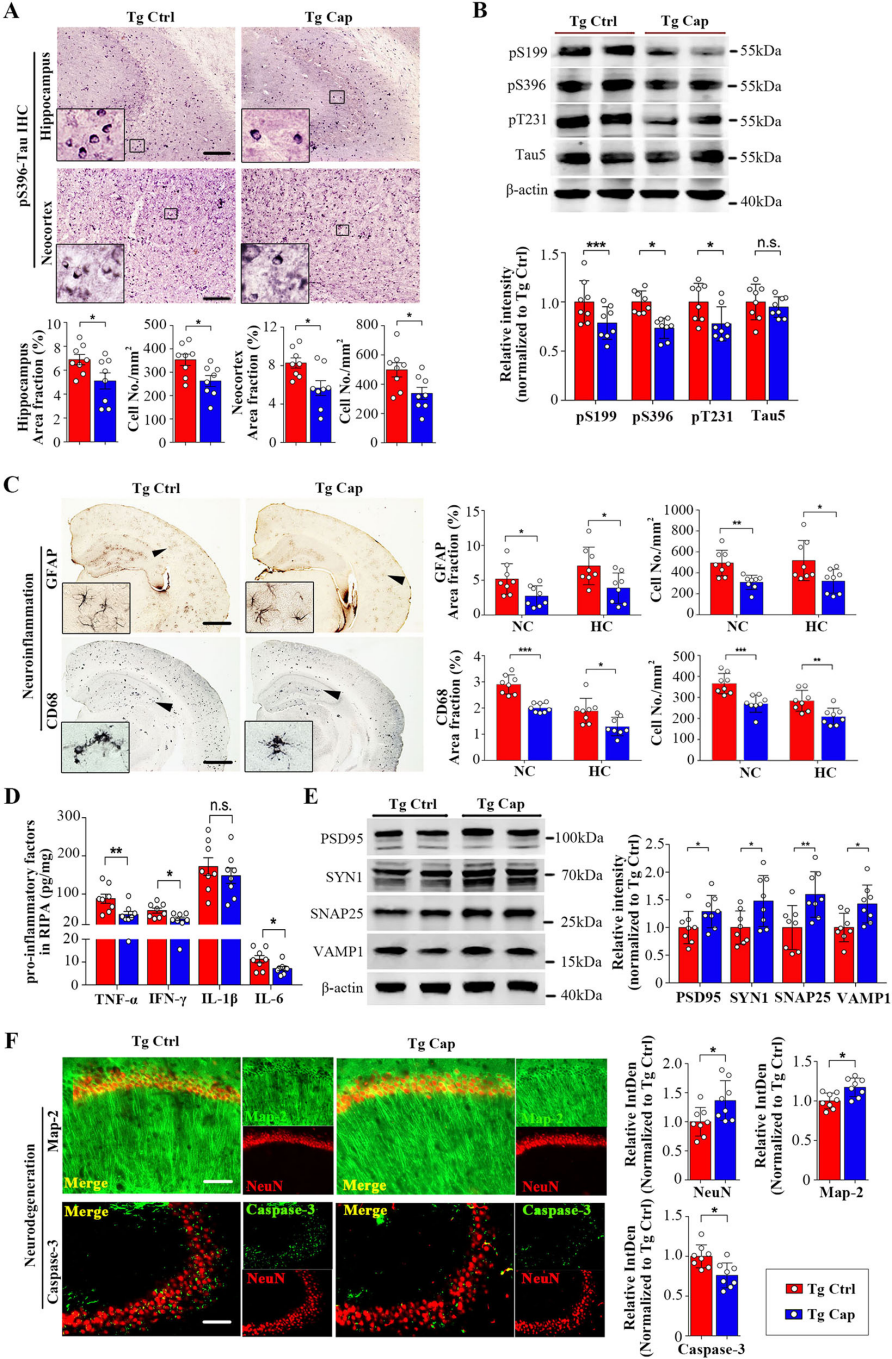
Capsaicin attenuates AD-type pathologies in APP/PS1 mice. **a** Representative images of PS396 immunohistochemical staining and quantitative analysis of pS396-positive staining in the neocortex and hippocampus. *Insets* show the representative morphology at higher magnification. *Scale bar*, 50μm. **b** Western blots and quantification of phosphorylated tau at multiple sites in brain homogenates. **c** Immunostaining and quantification of activated microgliosis (CD68) and astrocytosis (GFAP) in neocortex and hippocampus. *Insets* show the representative morphology at higher magnification. *Scale bar*, 500μm. **d** ELISA assays of proinflammatory factors in brain homogenates of Tg Ctrl and Tg Cap mice. **e** Western blot and quantitative analysis of synapse-related proteins in brain homogenates. **f** Representative images and quantification of fluorescence intensity (relative integrated density) of neurons and dendrites in the CA1 region of the hippocampus stained with anti-NeuN and anti-MAP-2 immunofluorescence and neuronal apoptosis in the CA3 region stained with activated caspase-3 immunofluorescence. *Scale bar*, 20μm. *N* = 8 per group. Data are presented as mean ± SEM. Student’s *t* test, two-sided. *p < 0.05, **p < 0.01, ***p < 0.001. Tg APP/PS1 transgenic mice, CapCapsaicin, Ctrl Control, IntDen integrated density.

We investigated whether capsaicin could affect other AD-type pathologies in the brain of APP/PS1 mice. Capsaicin significantly attenuated hyperphosphorylation of tau in different brain regions and different phosphorylation sites. Phospho-Tau (pS396)-positive neurons in both neocortex and hippocampus were significantly decreased in the brain of capsaicin-treated mice compared to controls (Fig. 4a). The levels of tau phosphorylation at multiple epitopes, including the Ser199 (pS199), pS396 and Thr231, were reduced in the capsaicin group, whereas total tau (tau5) showed no significant differences between the two groups (Fig. 4b). Neuroinflammation was significantly ameliorated in the brain of capsaicin-treated mice compared with APP/PS1 controls, as reflected by decreased levels of activated microglia and astrocytes, and reduced levels of proinflammatory factors, including TNF-α, IFN-γ, and IL-6 (Fig. 4c, d). Capsaicin also attenuated neurodegeneration in APP/PS1 mice. Immunoreactivities of synapse-related proteins, including PSD95, SYN1, SNAP25 and VAMP1, were significantly elevated in the brain of capsaicin-treated APP/PS1 mice (Fig. 4e). Compared with APP/PS1 control mice, the capsaicin-treated mice displayed an increased fluorescence intensity of staining for NeuN and Map-2 and a decreased intensity of activated caspase-3 in the hippocampus (Fig. 4f). Taken together, these findings suggest that capsaicin protects against tau hyperphosphorylation, neuroinflammation and neurodegeneration in the brain of APP/PS1 mice.

## Discussion

**Fig. 5:**
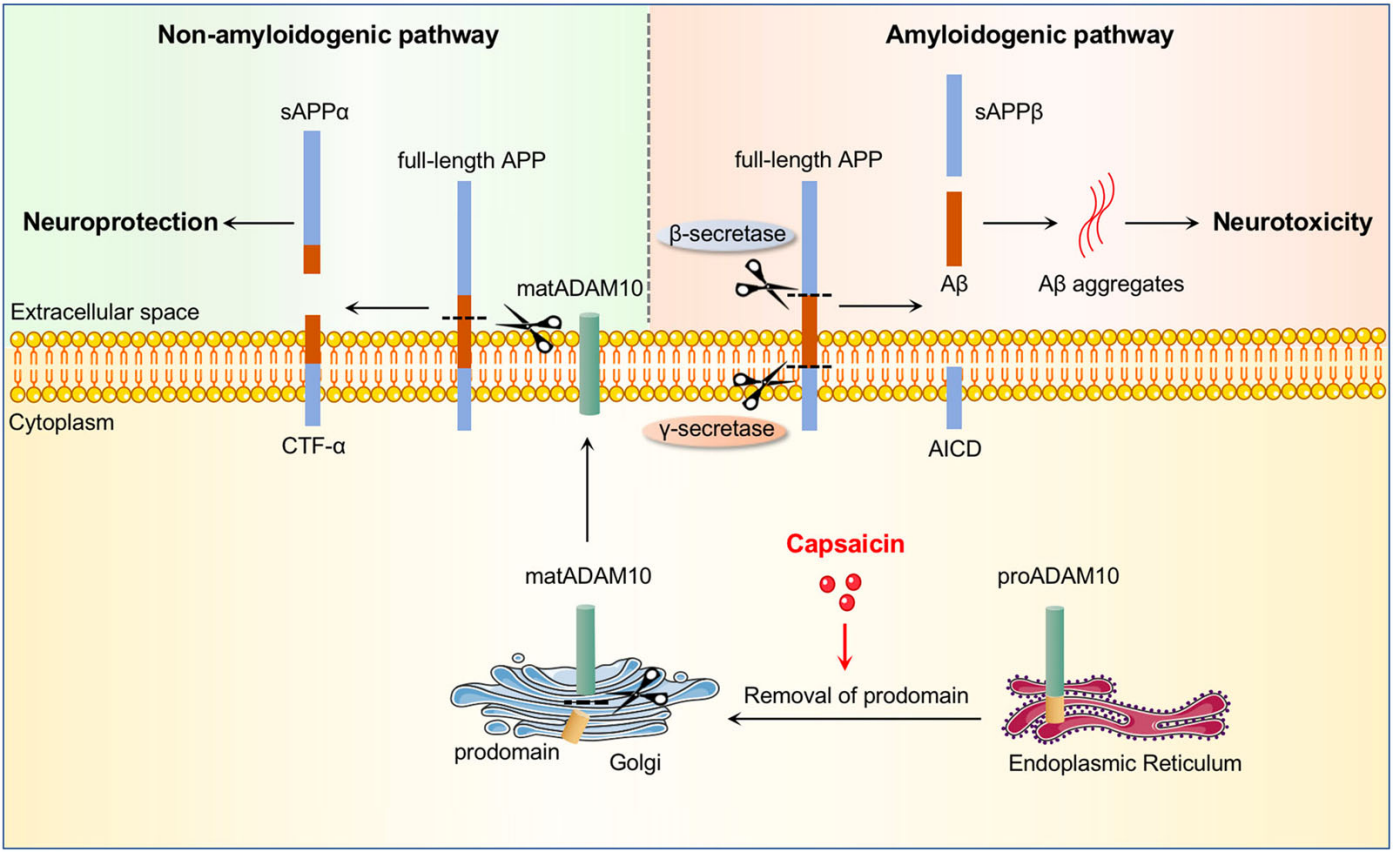
Schematic diagram of capsaicin inhibiting Aβ generation. APP processing includes non-amyloidogenic and amyloidogenic pathways. In the amyloidogenic pathway, Aβ is generated by the sequential cleavage of APP by β- and γ-secretases. In the non-amyloidogenic pathway, APP is cleaved by α-secretase within the Aβ domain, which precludes Aβ generation. Increased α-secretase activity competitively inhibits amyloidogenic processing of APP and decreases Aβ production. ADAM10 is the major α-secretase responsible for ectodomain shedding of APP in the brain. proADAM10 is expressed in endoplasmic reticulum and processed in Golgi where the prodomain of proADAM10 is removed and mature ADAM10 (matADAM10) is generated; then matADAM10 is transported to the plasma membrane and executes catalytic functions. Capsaicin promotes the maturation of ADAM10, thereby shifts APP processing from amyloidogenic pathway to non-amyloidogenic pathway and precludes Aβ generation.

The aetiology of AD is multifactorial, and several potentially modifiable risk factors and protective factors have been identified. Dietary patterns have been linked with the risk of AD^26–29^. Healthy diet, as one component of the approach of multidomain lifestyle interventions, has showed beneficial effects on the prevention of cognitive impairment in several trials^1,30–32^. In the present study, we showed that dietary capsaicin, the major pungent ingredient in chili peppers, effectively reduced brain Aβ burden, attenuated neurodegeneration and improved cognition in APP/PS1 mice; our in vivo and in vitro studies indicated that capsaicin inhibited Aβ generation via promoting non-amyloidogenic processing of APP. Overproduction of Aβ plays a key role in the pathogenesis of AD^33,34^. Aβ is generated by the sequential cleavage of APP via β- and γ-secretases. Alternatively, APP can be cleaved by α-secretase within the Aβ domain, which precludes Aβ generation. Increased α-secretase activity competitively causes decreased β-secretase processing of APP and Aβ production^35^. ADAM10 is the major α-secretase responsible for ectodomain shedding of APP in the brain^36,37^. In the present study, we found that capsaicin treatment increased the maturation of ADAM10 and thereby precluded Aβ generation (Fig. 5). Additionally, capsaicin also upregulated the levels of PPARα, which could activate ADAM10-mediated proteolysis of APP^25^, suggesting that capsaicin might activate ADAM10 via upregulating PPARα.

In AD patients, ADAM10 activity has been reduced in both CSF and brain samples^38,39^. Recently, several rare mutations have been associated with late-onset AD (LOAD) in the prodomain of ADAM10, and two mutations promote amyloid pathology by diminishing α-secretase activity^40^. This year, a genome-wide association study (GWAS) identified a common variant in ADAM10 which was associated with increased AD risk^41^, which has been confirmed in Han Chinese population^42^. These findings suggest that decreased levels and activity of ADAM10 are involved in AD pathogenesis, and ADAM10 is a promising therapeutic target^43^. The overexpression of ADAM10 can reduce Aβ levels and prevent its deposition in plaques, as well as rescue cognitive defects in animals^35^. Several drugs intended as indirect α-secretase activators have progressed to the clinical trial stage for AD, such as GABA receptor modulator etazolate, 5-HT4 agonist PRX-03140, and a polyphenolic compound from green tea, epigallocatechin-gallate, but no results have been published to date (NCT00880412, NCT00693004, NCT00951834). At present, no direct α-secretase or ADAM10 activators are used for AD treatment. Our findings suggest that capsaicin is a natural ADAM10 activator and shows potential to attenuate amyloid pathology and protect against AD. However, further mechanism studies, such as inhibition or knockout of ADAM10, are needed to demonstrate ADAM10-mediated inhibition effects of capsaicin on Aβ generation.

TRPV1 is known as capsaicin receptor. Recent studies has reported that genetic upregulation of TRPV1 attenuated Aβ burden in brain of AD model mice and rescued memory decline and Aβ-induced neuronal function and network impairment in vivo and in vitro^44,45^, although controversial report exists^46^. In our present study, we did not detected significant differences in TRPV1 expression between mice received capsaicin-rich diet and those received standard diet. TRPV1 is a calcium-permeable non-selective cation channel, it is possible that dietary capsaicin activates TRPV1 via altering its spatial conformation, but doesn’t affect its expression; another possible explanation is that the inhibition effects of capsaicin on Aβ generation is not, at least not completely, dependent on TRPV1 activation.

Our previous cohort study suggests that chili consumption is protective against cognitive impairment in subjects aged 40 years and older^14^. A very recent paper suggests that higher chili intake is associated with worse memory decline in an open cohort study conducted in China^47^. However, this study had big biases as it used the objective self-reported memory decline, and the subjects who had higher consumption of chili food were lower educated. Our present findings further support the protective effects of chili consumption on cognition. In previous studies, it has been reported that capsaicin could improve cognitive performance in wild-type mice or rats but with controversy^45,48,49^, all studies did not find obvious adverse effects of capsaicin administration. Further studies are required to make clear whether capsaicin can enhance memory in wild-type animals or healthy humans. Additionally, capsaicin has been reported to ameliorate tau changes and behaviour impairments in Aβ-independent pathway in stress- or drug-induced nonspecific AD models which only display tau pathologies and cognitive impairment but no Aβ plaques^50–52^. In our present study, APP/PS1 transgenic AD mouse model was used. In this mouse model, tau hyperphosphorylation is secondary to Aβ pathologies. Therefore in our study, the reduction of tau phosphorylation and pathologies should be mainly resulted from the reduction of Aβ production due to capsaicin. Previous and our present studies suggest that capsaicin protects against AD by targeting multiple pathogenesis of disease. Contrast to our results, another study showed that subcutaneous injection of capsaicin enhanced the level of membrane-bound APP in Sprague–Dawley rats^53^. In this study, capsaicin was given by subcutaneous injection twice in two days which is an acute or short-term treatment, but in our present study and studies mentioned above, capsaicin was given by oral administration or intragastric infusion for a chronic long-term treatment. This may explain the different results among these studies. The dose of capsaicin used in the current study (30 mg/kg) is higher than daily consumption of human. It has been reported that low-dose of capsaicin (1–2 mg/kg) exerted antiepileptic effects^54,55^, whereas high-dose of capsaicin (10–120 mg/kg) exerted proepileptic effects in animal models^56,57^. In our study, we did not find the occurrence of obvious seizures or increased mortality in capsaicin-treated mice. The safe and effective doses of capsaicin for AD in both humans and animals need to be determined in future studies.

Capsaicin, as a natural component of spicy food, has potential advantages as an AD intervention strategy. Considering that chili peppers have been a vital part of culinary cultures worldwide and have a long history of application for flavouring, they are feasible to utilized for AD prevention. In addition, capsaicin is a potential therapeutic molecule for various human diseases, such as obesity, cardiovascular diseases, hypertension, and atherosclerosis, which are established risk factors for AD^16,17,58^. Taken together, the current and previous findings suggest capsaicin may prevent AD by targeting multiple pathways that drive the pathogenesis of AD.

In conclusion, we uncovered an application of capsaicin, the pungent ingredient in chili peppers, as a promising therapy for AD. Capsaicin has moved towards clinical applications and is used currently in topical creams and gels to relieve intractable neuropathic pain, uremic pruritus, and rheumatoid arthritis. Our findings warrant future clinical trials on chili peppers or capsaicin as dietary supplementation for the prevention of AD. Meanwhile, global epidemiological studies are worthy to explore the association between capsaicin-rich diet and AD prevalence.

## Supplementary information

Supplementary information
