# Supplementary material for: Capsaicin consumption reduces brain amyloid-beta generation and attenuates Alzheimer’s disease-type pathology and cognitive deficits in APP/PS1 mice
Source: Transl Psychiatry. 2020 Jul 13;10:230. doi: 10.1038/s41398-020-00918-y (PMC7359297; doi:10.1038/s41398-020-00918-y)
Supplement: Supplementary file 1 — Supplementary information [file 41398_2020_918_MOESM1_ESM.docx]

**Capsaicin consumption reduces brain amyloid-beta** **generation and attenuates Alzheimer’s disease-type pathology and cognitive deficits in APP/PS1 mice**

Running head: Capsaicin protects against Alzheimer’s disease

Jun Wang, PhD ^1,#,*^, Bin-Lu Sun, MD ^1,#^ , Yang Xiang, PhD ^2^, Ding-Yuan Tian, MD ^1^, Chi Zhu, PhD ^1^, Wei-Wei Li, PhD ^1^, Yu-Hui Liu, PhD ^1^, Xian-Le Bu, PhD ^1^, Lin-Lin Shen, PhD ^1,3^, Wang-Sheng Jin, PhD ^1^, Zhen Wang, PhD ^4^, Gui-Hua Zeng, MS^1^, Wei Xu, PhD ^5^, Li-Yong Chen, PhD ^4^, Xiao-Wei Chen, PhD ^6^, Zhian Hu, PhD ^7^, Zhi-Ming Zhu, PhD ^8^, Weihong Song, PhD ^9^, Hua-Dong Zhou, PhD ^1^, Jin-Tai Yu, PhD ^10^ and Yan-Jiang Wang, PhD ^1,11,12,*^

**Supplementary Figures**


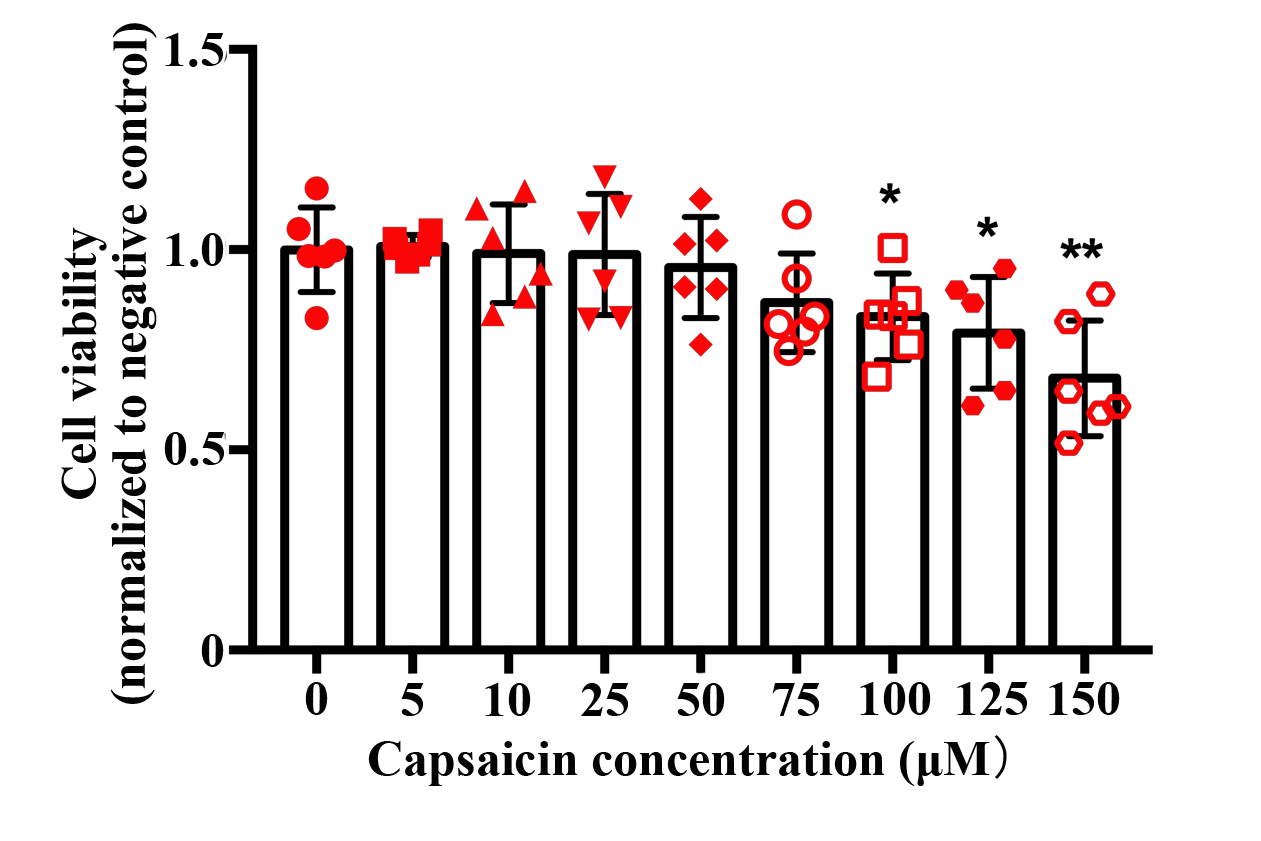


**Supplemental Fig 1. Cell viability after capsaicin treatment.** SH-SY5Y-APP695swe cells were treated with or without an increasing concentration of capsaicin for 24 h. Cell viability was measured by MTT assay. Capsaicin impaired cell viability at concentrations >75μM. N=6, mean±SEM. Student’s *t*-test, **p*<0.05, ***p*<0.01, compared with negative control (0 μM capsaicin).


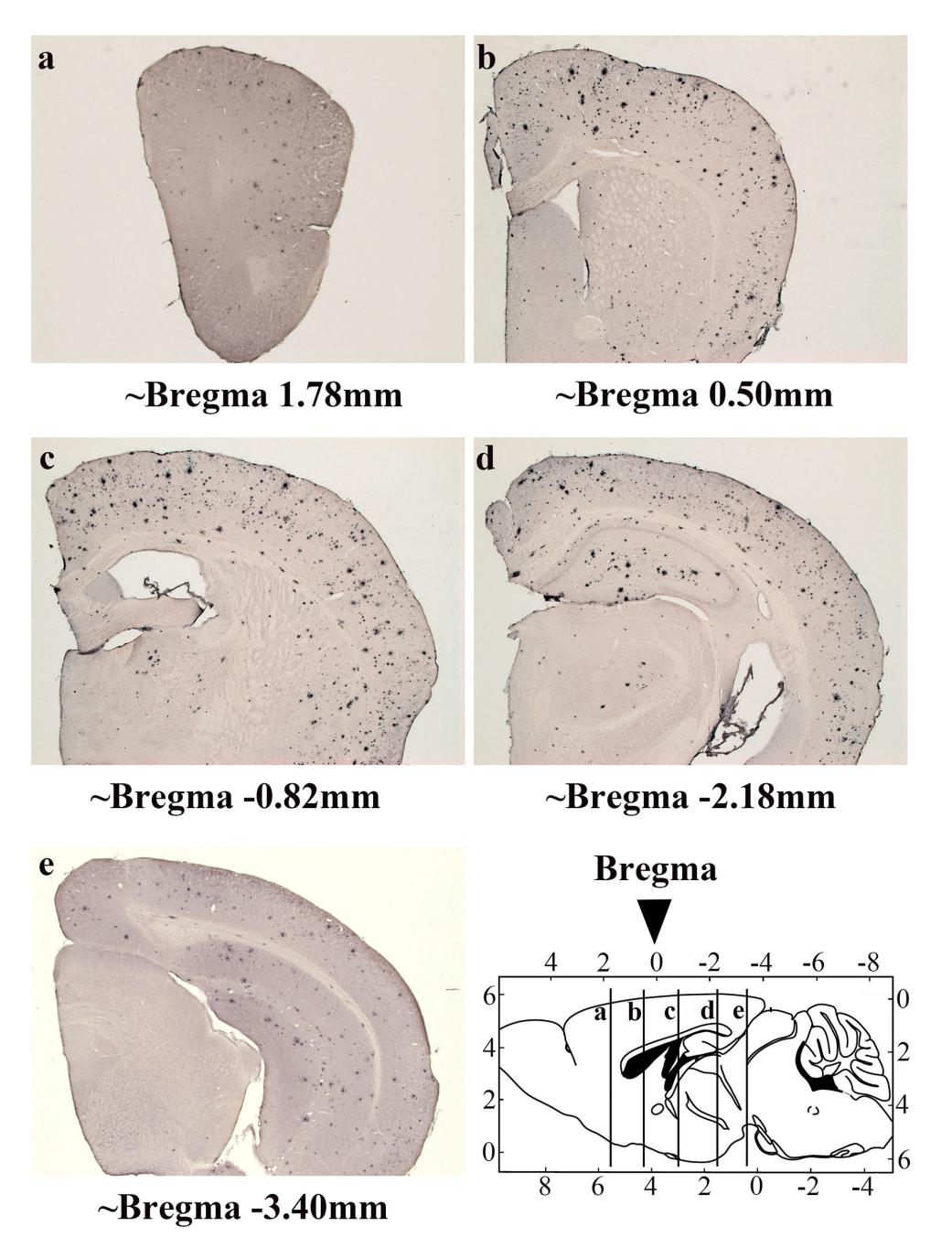


**Supplemental Fig 2.** **Location of five representative sections used for histological analysis.** A series of five equally spaced tissue sections (~1.3 mm apart) spanning the entire brain were selected for histology in our present study.


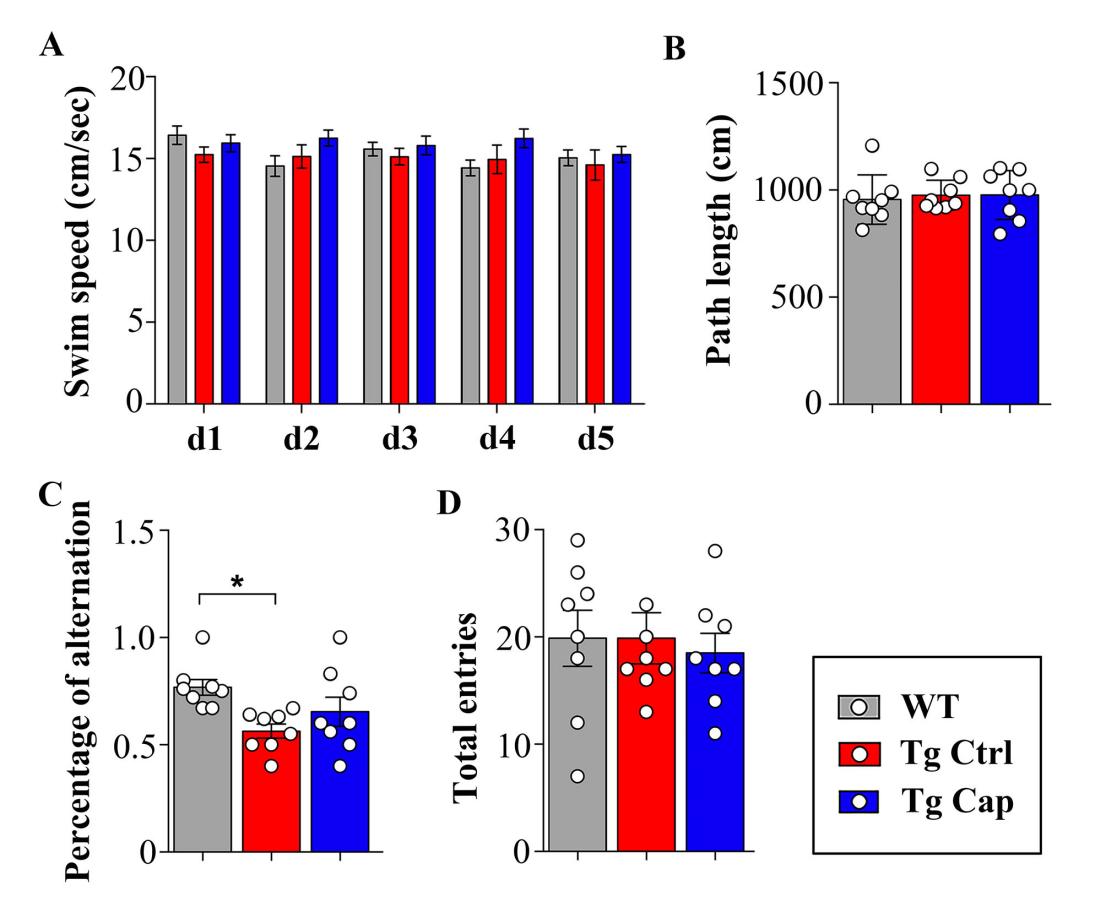


**Supplemental Fig 3. Morris water maze test and spontaneous exploration test in prevention trial.** (A) To exclude differences in motor performance among groups, (A) swim speed during platform trials and (B) mean path length to the platform during the probe trials were analyzed. No differences were detected among three groups. (C) In spontaneous exploration test in Y-maze, Tg ctrl mice display worse performance in the spontaneous exploration test reflected by reduced percentage of alternation, capsaicin seems to rescue this deficit but it did not reach statistically significance. (D) There were no significant differences in total entries into all three arms during spontaneous exploration test. N=8 per group. Values are presented as the mean±SEM. *p<0.05, two-sided.
